# The Relation of Obesity-Related Hormonal and Cytokine Levels With Multiple Myeloma and Non-Hodgkin Lymphoma

**DOI:** 10.3389/fonc.2018.00103

**Published:** 2018-04-16

**Authors:** H. Dean Hosgood, Marc J. Gunter, Neil Murphy, Thomas E. Rohan, Howard D. Strickler

**Affiliations:** ^1^Department of Epidemiology and Population Health, Albert Einstein College of Medicine, Bronx, NY, United States; ^2^Section of Nutrition and Metabolism, International Agency for Research on Cancer, World Health Organization, Lyon, France

**Keywords:** non-Hodgkin lymphoma, multiple myeloma, inflammation, hyperinsulinemia, sex hormones

## Abstract

This article presents the first detailed overview of the mechanisms that may underlie the relation of obesity with B-cell non-Hodgkin lymphomas (NHLs) and multiple myeloma (MM). Epidemiologic studies, including meta-analyses of prospective cohorts, have reported that the risks of NHL and MM are significantly increased in obese, relative to normal weight, women and men. Accumulating experimental and clinical evidence suggests that inflammatory cytokines, hyperinsulinemia, and sex hormones could play a role in the association of obesity with B-cell NHL and MM carcinogenesis. There is, however, a paucity of data published from appropriate large prospective cohort studies, and studies concurrently measuring these correlated factors, to formally determine the likely biologic factors driving the relationship of obesity with NHL and MM. Additional strengths and weaknesses of the current literature, as well as study design issues that need to be considered in conducting these studies, such as the exclusion of type 2 diabetics or postmenopausal women using hormone therapy, are discussed.

## Introduction

Hematopoietic malignancies originating from B-cell lymphocytes include each of the major non-Hodgkin lymphoma (NHL) subtypes, namely, diffuse large B-cell lymphoma (DLBCL), follicular lymphoma (FL), and chronic lymphocytic leukemia (CLL), as well as multiple myeloma (MM). NHL and MM are the two most common hematologic malignancies in the United States, with a combined total of approximately 100,000 new cases per year (1). However, there are few well-established risk factors for B-cell NHL beyond male gender, African ancestry, family history, viral infection and diminished immune function, and African ancestry and family history for MM (2–5).

A number of studies, including several meta-analyses, have found significant positive associations between obesity and risk of NHL and MM, adding to the well-known associations of obesity with risk of several common solid tumors (6–8). The International Agency for Research on Cancer (IARC) has deemed the strength of evidence linking adiposity with MM and DLBCL to be sufficient and suggestive for all NHL (9). Accumulating experimental and clinical evidence suggests that inflammatory cytokines, hyperinsulinemia, and sex hormones could play a role in the association of obesity with B-cell NHL and MM carcinogenesis. To date, the associations of these factors with NHL and MM risk have not been concurrently evaluated in appropriate large, long-term prospective cohort studies. Such studies are needed to elucidate the association of these factors with risk of hematopoietic malignancies, especially as circulating levels of these factors are correlated with each other. Furthermore, most of the existing epidemiological studies have not accounted for type 2 diabetes or the use of diabetes medications, even though the biologic connection between obesity and these molecular pathways may be altered by diabetes treatment and potentially by type 2 diabetes itself. Likewise postmenopausal women using hormone therapy (HT) should be excluded from such studies, as oral estrogens alter circulating levels of many of these obesity-related factors. Furthermore, stronger and more consistent associations with cancer risk would be expected if the obesity-related downstream molecular pathways underlying obesity’s relation with MM and B-cell NHL were directly assessed as exposure variables rather than adiposity itself. While a number of review papers regarding obesity-related cancer have been published (10–18), to the best of our knowledge this article represents the first detailed overview of the mechanisms that may underlie the relation of obesity with hematologic malignancies.

## The Relationship of Obesity with B-Cell Lymphomas and MM

The relation of hematological malignancies with obesity has been evaluated extensively (6, 19), including in a number of large cohort studies (Table 1). In 2007, for example, prospective analysis of the National Institutes of Health–American Association of Retired Persons (NIH–AARP) Diet and Health Study (*n* = 473,984) observed that those with a body mass index (BMI) ≥ 35 kg/m^2^ had an increased risk of NHL [relative risk (RR) = 1.29; 95% confidence interval (CI) = 1.02–1.64] compared with individuals with a normal BMI (i.e., 18–25 kg/m^2^) (19). In 2011, Larsson and Wolk critically reviewed prospective studies of the NHL–obesity association (6). Among the 16 NHL studies (*n* = 17,291 cases) that had been conducted up until that time, a meta-analysis showed that a 5 kg/m^2^ increase in BMI was associated with a 7% increased risk of NHL (RR = 1.07; 95% CI = 1.04–1.10), which was primarily restricted to DLBCL (RR = 1.13; 95% CI = 1.02–1.26) (6). However, there was no statistically significant heterogeneity in the subtype-specific associations, and thus the association of obesity with NHL may include additional NHL subtypes. These findings did not differ by gender and were not affected by adjustment for physical activity, or whether height and weight were directly measured or provided by self-report. Since publication of Larsson and Wolk’s review, an additional four prospective cohort studies have evaluated the association between obesity and NHL risk (20–23). In the Women’s Health Initiative (WHI) cohort study, with 1,123 incident NHL cases (in 158,975 postmenopausal women), Kabat et al. observed that women in the highest quartile of weight and of BMI at age 18 had an increased risk of NHL (21). Furthermore, in the Cancer Prevention Study II (CPS II), Patel et al. observed an increased risk of NHL associated with obesity (≥30 versus 18.5– <25 kg/m^2^) in their investigation with 2,074 incident NHL cases (in 152,423 men and women) (23). The other two recent prospective studies also found significant positive associations between obesity and NHL risk (20, 22). However, out of a current total of 22 prospective cohort studies, only 3 studies took into account the presence or absence of diabetes in the analyses (24–26). These studies adjusted their analyses for diabetic status (24–26), raising the possibility that many of the reported associations, while statistically significant, may be attenuated due to the inclusion of type 2 diabetics and pre-diabetics (see Section “Design Considerations for Studies of Obesity-Related Hormones and Adipocytokines in the Etiology Studies of Cancer” regarding study design considerations). Only 2 of the 22 studies accounted for the use of HT or menopause status (25, 27), raising similar considerations of possibly attenuated associations. After adjustment for both diabetes and HT, Cerhan et al observed a 2.7-fold increased risk (95% CI = 1.2–6.0) of B-CLL and high BMI (≥28.3 kg/m^2^) at age 50 (25).

**Table 1:** Characteristics of prospective cohort studies evaluating the risk of non-Hodgkin lymphoma (NHL) associated with obesity.

Country	Cohort size	Number of NHL cases	% Cohort females	% Cohort males	Diabetic status addressed?	Exogenous hormones addressed?	Reference
Australia	40,909	310	59	41	No	No	(28)
Austria	145,931	148	54	46	No	No	(29)
Denmark, France, Germany, Greece, Italy, Netherlands, Norway, Spain, Sweden, and United Kingdom	371,983	1,219	62	38	Models were adjusted for diabetes	No	(24)
Israel	2,352,988	4,201	40	60	No	No	(22)
Japan	94,547	205	52	48	No	No	(30)
Korea	781,283	190	0	100	No	No	(31)
Norway	2,000,334	8,512	52	48	No	No	(32)
Sweden	68,786	89	51	49	No	No	(33)
Sweden	362,552	1,077	0	100	No	No	(34)
Sweden and Finland	70,067	290	53	47	Models were adjusted for diabetes	No	(26)
The Netherlands	120,852	517	51	49	No	No	(35)
United Kingdom	1,222,630	1,509	100	0	No	Models were adjusted for hormone therapy (HT) use, years since menopause	(27)
United States	88,410	199	100	0	No	No	(36)
United States	37,931	261	100	0	Models were adjusted for diabetes	Models were adjusted for HT use	(25)
United States	473,984	1,381	40	60	No	No	(19)
United States	142,982	1,264	50	50	No	No	(37)
United States	152,423	2,074	53	47	No	No	(23)
United States	46,390	635	0	100	No	No	(20)
United States	121,216	574	100	0	No	No	(38)
United States	158,975	1,123	100	0	No	No	(21)

Multiple myeloma and obesity have similarly been studied in a number of prospective cohort studies (39). The NIH-AARP study observed that each 5 kg/m^2^ increase in BMI was associated with a 10% increased risk of MM (hazard ratio = 1.10; 95% CI = 1.00–1.22) (40), and Wallin and Larsson’s meta-analysis found a significantly increased risk of MM associated with being overweight (RR = 1.12, 95% CI = 1.07–1.18) or obese (RR = 1.21, 95% CI = 1.08–1.35), based on 15 cohort studies (39). No evidence of gender differences in these associations was observed, and none of the included studies accounted for diabetic status or HT use, either through statistical adjustment or inclusion/exclusion criteria. A recent pooled analysis in the International Multiple Myeloma Consortium (IMMC), which involved eight case–control studies, found that a 5 kg/m^2^ increase in BMI was associated with a 9% increased risk of adult MM [odds ratio (OR) = 1.09, 95% CI = 1.04–1.14] (41), but only when individuals were overweight or obese in both early and later adulthood, suggesting that risk is influenced only by persistently high adiposity.

As mentioned earlier, IARC has deemed the strength of evidence linking adiposity with MM and DLBCL to be sufficient, and suggestive for all NHL (9). The major focus of future investigation increasingly needs to be on the molecular mechanisms underlying the association of obesity with MM and NHL (especially DLBCL).

## The Relationship of Inflammatory Cytokines with B-Cell Lymphomas and MM

Obesity is correlated with chronically high levels of several cytokines (produced primarily by adipose tissues), which play a role in chronic inflammatory states. These cytokines include tumor necrosis factor alpha (TNFα), IL6, IL10, as well as C-reactive protein (CRP). In relation to CRP, recent data suggest that CRP may have pleomorphic immune activity in addition to its well-known role as a biomarker of inflammation (8). Dysregulation of the immune system is a hallmark of NHL, and laboratory studies of cytokines and hematopoietic malignancies have particularly focused on TNFα given its pivotal role in initiating inflammatory responses. TNFα regulates the immune response through activation of the nuclear factor kappa beta transcription factor, leading to the production of pro-inflammatory cytokines, including IL6 (42), which in turn can induce B-cell production of IL10 (43). Moreover, each of these cytokines promotes B-cell proliferation and has antiapoptotic activity (44–46). Additional members of the TNF superfamily include CD27 and CD30, which are expressed on B lymphocytes and promote lymphocyte survival and proliferation (47, 48). Experimentally, TNFα has also been shown to act as a tumor growth factor for chronic B-cell malignancies (49), promoting the survival and proliferation of malignant cells (44, 45).

The relationship between cytokine levels and risk of B-cell lymphoma has been quantified in several large, well-established prospective cohorts including WHI (50, 51), the Prostate, Lung, Colorectal, and Ovarian (PLCO) Cancer Screening Trial (52, 53), the European Prospective Investigation into Cancer and Nutrition Study (EPIC) (54), and the Multi-Ethnic Cohort (MEC) (55). WHI evaluated 11 cytokines (IL1b, IL2, IL4, IL5, IL6, IL10, IL12, IL13, TNFα, IFNc, and GM-CSF) and found that subsequent risk of incident B-cell NHL in postmenopausal women (491 cases, 491 controls) was associated primarily with high levels of TNFα and IL10: ORs per doubling in serum cytokine concentration were 1.22 (95% CI = 1.07–1.38) for TNFα and 1.09 (95% CI = 1.04–1.15) for IL10 (51). MEC reported similar findings (55). Selected cytokines (IL4, IL6, IL10, and TNFα) and other soluble markers of immune activation [soluble TNF receptor 1 (sTNF-R1), sTNF-R2, CRP, and sCD27] were measured in PLCO in a study of 297 incident NHL cases and 297 individually matched controls (52), finding NHL risk in this study was positively associated with sTNF-R1 (*P*_trend_ = 0.02) and sCD27 (*P*_trend_ < 0.0001) (52). A second study in PLCO showed positive associations of NHL with B-cell-attracting chemokine 1 (*P*_trend_ = 1.0 × 10^−6^), sTNF-R2 (*P*_trend_ = 1.1 × 10^−6^), and soluble vascular endothelial growth factor receptor 2 (*P*_trend_ = 0.0005) (53). Consistent with these results, positive associations of sCD27 and sCD30 with risk of NHL were recently also reported by the Alpha-Tocopherol, Beta-Carotene Cancer Prevention (ATBC) Study (56) and WHI (50). Overall, it would appear that relatively high levels of prediagnostic pro-inflammatory cytokines increase risk of NHL development. However, mediation analyses to estimate the proportion of the obesity–NHL that may be causally associated with inflammatory factors is warranted.

For MM, in contrast, there remains a paucity of similar studies of cytokines and CRP (57, 58). Furthermore, no studies of the aforementioned inflammatory factors and either NHL or MM concurrently, robustly controlled for additional obesity-related hormonal factors. Thus, despite the strong and consistent data appearing to link inflammatory cytokines with NHL, the extent to which the cytokine–NHL relationship might be explained by other correlated obesity-related hormonal factors remains unknown.

## The Relationship of Insulin and the Insulin-Like Growth Factor (IGF) Axis with B-Cell Lymphomas and MM

The relation of obesity, type 2 diabetes, and a sedentary lifestyle, with risk of several solid tumors, has led to the hypothesis that high insulin levels may increase risk of cancer (7, 8, 41, 59–62). In addition to its well-known metabolic activity, insulin is a growth factor for a wide range of tissues and in fact plays a significant role in normal organogenesis during fetal development. Insulin shares approximately 40% amino acid sequence homology with IGF-I, the primary mediator of the growth effects of growth hormone. Binding of insulin or IGF-I to their own receptors can promote cell proliferation by activation of the same two downstream pathways: the p21/ras/mitogen-activated protein kinase and phosphatidyl inositol-3 kinase (PI-3K) signaling pathways. Consistent with this, genes that underlie the PI-3K/AKT pathway are often mutated in several forms of NHL and Hodgkin lymphoma, resulting in high tumor tissue levels of phosphorylated AKT (a major oncogenic effector of this pathway) (63–65), whereas RAS/RAF mutations are common in MM (66). Insulin may also increase bioactive IGF-I levels by lowering production or increasing proteolysis of certain IGF binding proteins (IGFBPs). However, levels of IGF-I are less consistently elevated in obese or diabetic patients than are insulin levels. Thus, it is insulin, and not IGF-I, that has generally been thought to play a major role in the relationship of obesity/diabetes with cancer (60). For example, strong positive associations between fasting insulin levels and postmenopausal breast cancer have been observed (67, 68) and found to largely explain the relationship of obesity with breast cancer (e.g., statistical adjustment for insulin but not sex hormones in multivariate models eliminated the relation of obesity with disease) (67, 69), whereas there are conflicting data regarding the association of circulating IGF-I and postmenopausal breast cancer (67, 70, 71).

In animal models, insulin has independent mitogenic/antiapoptotic activity, and overexpression of insulin receptors (IRs) can result in cell transformation and tumor cell proliferation *in vitro* (72). IRs are highly expressed in many human cancers (73–76), including NHL and MM cell lines (77). However, it is generally the IR-A isoform (which plays a major role in fetal development) that is disproportionately expressed in most tumors, and not the IR-B isoform (which plays a major role in metabolism) (78). As with their ligands, there is considerable homology between IRs and the IGF-I receptor (IGF-I), although binding of the alternative receptor does not meaningfully occur at physiologic levels. Nonetheless, both IR-A and IR-B can form hybrid receptors with IGF-IR, and these hybrids are reported to bind both insulin and IGFs (despite ongoing uncertainty regarding their relative affinities) (78). Laboratory data suggest that it is most likely the IR-A/IGF-IR hybrid receptor that plays the major role in insulin signaling and MM tumorigenesis (79). Less data of this type have been reported for NHL. However, there are limited human data regarding the relation of circulating insulin or IGF-I levels with NHL and MM. A prospective cohort study observed that females reporting adult-onset diabetes were at an increased risk of NHL if they used insulin (80), but to the best of our knowledge no studies have assessed endogenous insulin levels and NHL. One multicohort consortium study of MM risk and its relation with prediagnostic IGF-I, as well as IGFBPs 1 and 3 (IGFBP-1 and IGFBP-3), has been reported (81). In this study, C-peptide, a marker of insulin secretion, was used as a surrogate for insulin. Of the insulin/IGF axis proteins studied, only IGFBP-1 had a significant (positive) association with MM. However, a substantial portion of bloods tested were non-fasting samples, and recent data have shown that fasting versus postprandial levels of C-peptide differ to a similar degree as insulin levels, making non-fasting C-peptide results difficult to interpret (82).

Overall, we are unaware of any studies of the insulin/IGF axis and its role in NHL or MM development that have adequately addressed concerns regarding the use of non-fasting blood specimens, inclusion of type 2 diabetics (or pre-diabetics using diabetic medication) or inclusion of postmenopausal women using HT. Finally, there are two isoforms of the IR. While the IR-B isoform plays the primary role in metabolism, IR-A plays a major role in fetal development and organogenesis, and it is IR-A that has been most associated with cancer development in laboratory and clinical studies (78, 83). Several common cancers have been shown to overexpress IR-A (78, 84–86). To the best of our knowledge, however, no studies have examined the IR isoforms in relation to risk of NHL or MM.

## The Relationship of Sex Hormones with B-Cell Lymphomas and MM

Obesity strongly influences several aspects of sex hormone biology. Obesity and high insulin levels are associated with lower levels of sex hormone binding globulin (SHBG) (87). Furthermore, obesity increases production of estrogen in postmenopausal women (88, 89) due to the activity of aromatase (estrogen synthetase) related biosynthesis of estrogens from lipids in adipose tissue (90, 91), and increases production of testosterone in men (92). As such, obesity-related reduced SHBG levels and increased production of estrogen and testosterone can lead to greater free/bioactive estrogen and testosterone levels.

Laboratory models have shown that estrogen can promote the differentiation, proliferation, and survival of hematopoietic cells (93, 94), and more specifically the maturation, selection, and secretion of antibodies by B-cells (95). Briefly, B cells develop from hematopoietic stem cells (HSCs) that originate in the bone marrow (96). These early phase B-cells then migrate to the spleen where they undergo further development, becoming mature B-cells (97). The administration of estrogen to mice reduces the mitotic activity of Ref. (98) and the number of B-cell precursors in the bone marrow (99). Estrogen (either alone or in combination with progesterone) administered to both male and female mice without exceeding the physiological levels observed during pregnancy was shown to promote the self-renewal of HSCs, as well as mobilization of T1 B-cells to the spleen for additional development (100).

There is considerable biologic cross talk between sex hormone signaling and other obesity-related hormonal pathways associated with risk of NHL and MM (101). For example, (i) ER and IGF-IR expression are correlated (102–107), (ii) estrogen increases IGF-IR levels (108), (iii) IGF-I enhances ER responsiveness to estrogen (109), (iv) insulin decreases levels of SHBG (110), (v) insulin upregulates androgen secretion by the ovaries (110), and (vi) insulin increases ER expression and binding capacity (60). Recent evidence suggests that estradiol administration significantly increases single cytokine secretion, notably TNFα and IL4, and polyfunctionality of antigen-specific T cells (111).

Epidemiologic studies have also provided some support for a role for sex hormones in the etiology of NHL and MM. Increased gravidity and parity are thought to reduce lifetime exposure to estrogens and have been associated with reduced risk of NHL (112–116), as is the case for postmenopausal breast cancer (117). For example, the InterLymph study reported that high gravidity was associated with reduced risk of FL in 7 of the 13 case–control studies that were analyzed (118). Three of these studies also observed significantly reduced risk of NHL with increased parity (119–121), and/or for FL with gravidity (118). The prospective California Teachers Study observed a reduced risk of NHL associated with increasing number of pregnancies and full-term pregnancies (116), although not all cohort studies have found these associations (112, 115).

Commonly used oral HT in the US contains animal-derived estrogens, predominantly estrone, that has lower mitogenic activity than estradiol, and use of estrogen-alone HT has been associated with reduced risk of estrogen-driven cancers such as postmenopausal breast cancer (122). Consistent with this, DLBCL risk is reduced with use of oral estrogen-alone HT. While the number of cases of NHL and MM in the WHI clinical trial was too limited to analyze separately, a pooled analysis of 9 case–control studies (2,094 cases; 2,731 controls) observed that, compared with never users, postmenopausal women who had ever used HT (estrogen or estrogen/progesterone) were at decreased risk of DLBCL (OR = 0.66, 95% CI = 0.54–0.80) and potentially FL (OR = 0.82, 95% CI = 0.66–1.01) (123). A recent pooled analysis of case–control studies (*n* = 7; ~1,000 cases) in the IMMC evaluated reproductive factors and exogenous hormone use in relation to the risk of MM in women and observed protective, but non-significant, associations with parity and HT use (124).

Overall, these data support the largely unexplored hypothesis that the sex hormone axis may play a role in lymphoma and MM development, as well as also the importance of addressing the extensive cross talk and correlation between sex hormones and other obesity-related molecular risk factors for these cancers. To the best of our knowledge, however, there have been no studies of circulating endogenous sex hormones and risk of DLBCL, FL, CLL, or MM, in women or men.

## Design Considerations for Studies of Obesity-Related Hormones and Adipocytokines in the Etiology Studies of Cancer

The collective findings to date not only provide important initial evidence that specific obesity-related hormonal and cytokine alterations may influence the risk of B-cell hematologic malignancies but also help inform decisions regarding future clinical/epidemiologic study design. Several research concerns particular to this area of investigation were briefly mentioned earlier and should be addressed:

- *Fasting blood specimens* should be used for measuring all factors that may vary postprandially, which include not only insulin but also C-peptide (a biomarker of insulin secretion). C-peptide has often been used as a surrogate for insulin when fasting specimens were unavailable, under the widespread assumption that C-peptide values are less altered by time since the last meal. However, several recent studies have shown that insulin and C-peptide values vary to a similar extent postprandially (82). Likewise, IGFBP-1, free/bioactive IGF-I, SHBG, and possibly free/bioactive estrogen and testosterone, may vary postprandially and should all be measured using fasting blood specimens. Alternatively, time since last meal may be used to address these concerns, albeit, not as robust as using fasting samples.
- *HT use and type 2 diabetes status* should be taken into account in future studies. Oral estrogens exert a strong first pass effect on the liver that alters hepatic protein production and changes levels of multiple hormones as well as inflammatory cytokines. Similarly, exclusion of type 2 diabetics and prediabetics using diabetes medication may be important since use of these medications is largely intended to reduce/counteract insulin resistance and also affects other insulin/IGF axis proteins, and other metabolic hormones, thereby potentially severing the relation of obesity with the relevant molecular pathways involved in hematologic carcinogenesis. A precedent for this comes from a prior study of the relation of insulin with postmenopausal breast cancer in non-diabetic women, which showed that the association was much stronger following exclusion of HT users (67, 68). Regardless of whether HT use and type 2 diabetes are addressed in sensitivity analyses or through study design exclusion criteria (e.g., due to concerns regarding sample size), it is important to this field of research that these issues be addressed by all studies since it is a potential source of bias and of conflicting reports in the literature. In addition, the exclusion of individuals receiving testosterone treatment should be carefully considered.
- *Assessment of inter-related biomarkers* involves several considerations. First, sample size and power calculations must take these correlations into account, and if multiple comparisons are a concern then the *P*-value required for statistical significance may need to be reduced (e.g., using the Benjamini–Hochberg procedure) and false discovery rates should be evaluated. There may also be concerns regarding collinearity. However, collinearity is usually only relevant for very strong correlations (e.g., *r* > 0.8), and most of the hormones and cytokines discussed earlier have only moderate correlations. For example, among the strongest correlations is that between IGF-I and IGFBP-3, in the range of *r* = 0.3–0.5 (67). Additional analytical approaches, such as Mendelian randomization (125), may also help elucidate the roles of specific biomarkers. Finally, given how the insulin/IGF-1, inflammatory, and sex hormone pathways interrelate/cross talk, mediation analyses should be considered to further elaborate these associations. For example, counterfactual-based mediation analyses (126) can be carried out to refine the decomposition of the effects of sex hormones, inflammation, and insulin into the mediated effects versus the remaining direct effects.
- *Studies of hematologic malignancies* also require additional, special consideration. Given the heterogeneity of disease subtypes, and associated etiological factors, studies must be designed to include sufficient number of cases for any particular subtype to ensure that subtype-specific analyses have >80% power. Given the rarity of NHL and MM, pooled nested case–control studies leveraging prediagnostic samples would be most likely needed.

## Conclusion

Obesity is now an established risk factor for DLBCL and MM, and possibly additional B-cell lymphoma subtypes. Growing evidence suggests that several obesity-related molecular factors and downstream signaling pathways that have been increasingly shown to play a role in carcinogenesis of several common solid tumors may also play a role in NHL and MM. These risk factors include inflammatory factors, particularly IL10 and TNFα, as well as sCD27 and sCD30; metabolic factors, particularly insulin and the IGF axis; and endogenous sex hormones, particularly estrogen. To date, however, these associations have not been concurrently evaluated in robust study designs (i.e., prospective cohorts with sufficient follow-up), which are needed to determine the likely underlying etiologic mechanisms, especially given the correlation among many of the obesity-related circulating factors. Stronger and more consistent associations with cancer risk would be expected if the obesity-related downstream molecular pathways underlying obesity’s relation with MM and B-cell NHL are directly assessed as exposure variables rather than adiposity itself. Obesity is highly prevalent in the US and many other countries around the world. Understanding the molecular pathways which underlie the relation of obesity with NHL and MM is key to identifying biomarkers for risk stratification, as well as new biologic targets for chemoprevention or treatment, particularly given the increasing incidence of hematological malignancies in countries experiencing obesity epidemics (127).

## Key Messages

- Obesity has been associated with risk of B-cell non-Hodgkin lymphomas (NHLs) and multiple myeloma (MM).
- The underlying mechanisms for obesity-related hematopoiesis may include inflammatory cytokines, hyperinsulinemia, and sex hormones; however, evidence is either limited or has been conflicting.
- The current literature is challenged by studies not robustly accounting for hormone therapy use and type 2 diabetes status, as well as having limited power for NHL subtype-specific analyses.
- All potential associations need to be concurrently evaluated in robust study designs (i.e., large-scale prospective cohorts with sufficient follow-up time and prediagnostic fasting blood samples) to determine the likely underlying etiologic mechanisms, especially given the correlation among many of the obesity-related circulating factors.

## Author Contributions

HH, MG, NM, TR, and HS were responsible for critically evaluating the content of the manuscript and providing approval for the final version to be published.

## Conflict of Interest Statement

The authors declare that the research was conducted in the absence of any commercial or financial relationships that could be construed as a potential conflict of interest. The reviewer JS and the handling Editor declared their shared affiliation.
